# Disrupted functional connectivity in the hippocampal subregions of patients with migraine without aura: a functional study on mechanisms underlying migraine chronification

**DOI:** 10.3389/fneur.2026.1821399

**Published:** 2026-06-02

**Authors:** Qifang Feng, Wen Chen, Hongru Zhao, Di Geng, Xing Xiong, Lingling Dai, Chao Zheng, Jun Ke, Chunhong Hu

**Affiliations:** 1 Department of Radiology, The First Affiliated Hospital of Soochow University, Suzhou, China; 2 Department of Neurology, The First Affiliated Hospital of Soochow University, Suzhou, China

**Keywords:** chronic migraine, dynamic analysis, functional connectivity, hippocampus, magnetic resonance imaging, subregion

## Abstract

**Background:**

Despite increasing evidence indicating hippocampal dysfunction in migraineurs, they overlooked the functional differences among hippocampal subregions and mostly used static functional connectivity (sFC). This study aimed to combine sFC and dynamic functional connectivity (dFC) to investigate the role of hippocampal subregional functional changes in migraine chronification.

**Methods:**

Resting-state functional MRI scans were performed on 52 patients with episodic migraine (EM), 22 patients with chronic migraine (CM), and 67 healthy controls (HCs). The bilateral rostral hippocampus (rHipp) and caudal hippocampus were selected as regions of interest. Differences in sFC and dFC values between the hippocampal subregions and other brain regions among the three groups were compared through the analysis of the general linear model. Spearman partial correlation analyses were used to assess the relationship between FC and clinical parameters in the migraine cohort.

**Results:**

In the sFC analysis, both EMs and CMs showed reduced connectivity between the left rHipp and the left orbital part of medial frontal gyrus as compared with HCs. In the dFC analysis, both the CM and EM groups exhibited increased connectivity between the right rHipp and the right calcarine compared to HCs, and the CM group demonstrated decreased dFC between the aforementioned brain regions relative to the EM group. No significant correlations were found between sFC and dFC values and clinical characteristics in patients with EM and CM.

**Conclusion:**

Altered sFC and dFC between the rHipp and the orbital part of medial frontal gyrus and calcarine in CM patients may be associated with dysfunctions in emotion, cognition, and multisensory integration, providing new insights into mechanisms of migraine chronification.

## 1 Introduction

Migraine, ranked as the third most prevalent medical condition and the second most disabling neurological disorder worldwide, is characterized by moderate to severe headaches and frequently accompanied by nausea, vomiting, and sensitivity to light or sound (1, 2). Based on the attack frequency, migraines are typically classified into episodic migraine (EM) and chronic migraine (CM) (3). In comparison to EM, CM leads to greater disability rates, significantly diminished quality of life, increased social burden, and substantial economic losses, largely attributable to inadequate headache treatment and associated comorbid conditions (4). Consequently, it is essential to adequately manage individual migraine attacks and to reduce the risk of migraine chronification. Although the pathophysiology of migraine chronification is still unclear and debated, increasing evidence suggests that it may be linked to altered function and structure in certain brain regions (5, 6).

With the continuous advancement of neuroimaging technologies, the hippocampus has received widespread attention as a key structure for elucidating the pathophysiological mechanisms of migraine (5–7). As a multifunctional structure located in the limbic system, the hippocampus is involved in memory, pain processing, emotion regulation, and stress response (7). Prior studies have revealed that migraineurs especially those with EM and CM exhibited structural and functional alterations in the hippocampus, characterized by volumetric reduction and neural dysfunction (5, 7). Besides, the correlations between the hippocampus and both the stress response and neuroinflammation have been comprehensively reported (8, 9), while these regulatory mechanisms are implicated in the migraine chronification (10, 11). Consequently, we specially focused on the hippocampus, aiming to gain deeper insights into its involvement in the neurobiological mechanisms underlying migraine chronification.

Recently, accumulating studies have reported altered hippocampal brain connectivity in patients with migraine (12, 13), but the variability of these changes indicates considerable uncertainties and the necessity for further research. The possible reasons include differences in analytical methods, the grouping of EM and CM into a single migraine cohort, variations in sample selection, and other methodological factors. Among these, one important reason is that these studies often analyze the hippocampus as a unitary structure, potentially overlooking the functional differences in subregions of the hippocampus related to the disease (14). Characterized by intrinsic functional diversity, the hippocampus can be divided into rostral (anterior) and caudal (posterior) subregions along its dorsoventral axis with functional differences. The caudal hippocampus (cHipp) is crucial for spatial learning, while the rostral hippocampus (rHipp) is predominantly involved in mood and emotional regulation (15). Recently, studies have explored the functional heterogeneity within hippocampal subregions in certain disorders based on resting-state functional magnetic resonance imaging (rs-fMRI) (16, 17). Although neuroimaging studies have revealed altered hippocampal connectivity in migraine (5, 12), it remains unclear how specific hippocampal subregions contribute to these changes, especially in EM and CM, respectively. Considering the functional specificity of the hippocampus along the rostro-caudal axis, and that migraine chronification is accompanied by emotion- and spatial memory-related regulatory deficits (18, 19), we hypothesize that migraine patients, especially those with EM and CM, may exhibit rostro-caudal hippocampal subregion-specific features in hippocampal-related connectivity patterns. Specifically, the rHipp may be associated with aberrant connectivity related to emotion regulation, whereas the cHipp may be associated with aberrant connectivity related to spatial learning (15). These subregion-specific connectivity differences may help elucidate the neural mechanisms underlying migraine chronification.

In rs-fMRI studies, while FC has been widely used to identify and localize aberrant neural communication patterns between the hippocampus and other brain regions in migraine patients (12, 20), these investigations are all based on static functional connectivity (sFC), potentially missing the dynamic reorganization of brain regions. In contrast, the dynamic functional connectivity (dFC) enables tracking real-time spontaneous changes in brain activity across different brain states through advanced analytical techniques (21). Recent neuroscience studies have increasingly employed this technique to characterize brain alterations in various disorders, such as depression, obstructive sleep apnea, and Alzheimer’s disease (16, 17, 22). Indeed, it has been demonstrated that dynamic analysis reveals more migraine-related functional network abnormalities than traditional static analysis, and migraine chronification may be associated with dynamic dysfunctional connectivity patterns between sensory and cognitive networks (18). Additionally, both EMs and CMs are under dynamic states, with migraine chronification exhibiting reorganization of brain networks (23). As such, sFC may not adequately capture this complex changing process, whereas dFC can reveal the dynamic changes in connectivity patterns between brain regions and help to further reveal the underlying mechanisms of migraine chronification. However, little is known about migraine chronification-related dFC in hippocampal subregions. Based on these findings, we assumed that the differences in sFC and dFC observed in migraine patients between hippocampal subregions and other brain regions may be associated with migraine chronification.

This research was intended to systematically assess the sFC and dFC between the rostral-caudal hippocampal subregions and other brain regions among the CM, EM and HC groups. Furthermore, we then analyzed the correlations between differential FC values and the clinical characteristics of the migraine patients.

## 2 Materials and methods

### 2.1 Subjects and clinical assessment

A total of 141 participants were enrolled in the study, including 52 patients with EM, 22 patients with CM, and 67 healthy controls (HCs) with balanced age, gender, and years of education. All migraine patients were recruited from the Department of Neurology. The diagnosis of these patients was completed by a neurologist specialized in headaches, following the criteria of the International Classification of Headache Disorders (ICHD-3 beta) (3). The demographic and clinical data collected from all patients included age, gender, years of education, frequency of headaches, duration of the disease, and pain intensity assessed by the 10-point Visual Analogue Scale (VAS). To avoid any potential interference on fluctuations in the blood-oxygenation-level-dependent signal, patients were required to be free of migraine attacks and medications for at least 3 days prior to and 1 day after the MRI scanning. Participants were excluded if they met any of the following criteria: left handedness; migraine with aura; preventive or therapeutic migraine treatments or therapies within 3 days before the MRI scanning; with a history of preventive migraine treatments; any other neurological or psychiatric diseases, including depression, anxiety, impulsivity, cognitive diseases and post traumatic stress disorder; MRI contraindications; drug or alcohol abuse. All the subjects read and signed informed consents in accordance with the Declaration of Helsinki before participation, and the study was approved by the ethics committee of the First Affiliated Hospital of Soochow University.

### 2.2 MRI acquisition

MRI examinations were performed using a 3.0-Tesla MRI system (MAGNETOM Skyra, Siemens Healthcare, Erlangen, Germany) equipped with a 16-channel head and neck joint coil. Participants were instructed to remain calm and keep their eyes closed while staying awake, especially during the resting-state scanning. To minimize noise caused by head motion and scanner operation, foam padding and earplugs were used during the scanning. For each subject, a sagittal fast spoiled gradient recalled echo sequence was used to obtain a high resolution T1-weighted anatomic image with the following parameters: repetition time (TR) = 2,300 ms, echo time (TE) = 2.98 ms, flip angle (FA) = 9°, field of view (FOV) = 256 × 256 mm^2^, matrix = 256 × 256, slice number = 192, slice thickness = 1 mm. Participants with visible structural abnormalities or brain lesions, as assessed by two professional radiologists, were excluded from the study. Subsequently, the resting-state functional images were acquired with an echoplanar imaging sequence with the following parameters: TR = 2000 ms, TE = 30 ms, FA = 90°, FOV = 256 × 256 mm^2^, matrix = 64 × 64, slice number = 33, slice thickness = 4 mm, no intersection gap. A total of 240 volumes were collected during each functional run.

### 2.3 Image processing

Preprocessing of rs-fMRI data was performed using the Data Processing and Analysis for Brain Imaging (DPABI).^1^ Initially, we eliminated the first ten volumes to ensure stabilization, corrected slice timing to make the results approach the actual results at a specific point in time, and realigned images to mitigate head motion artifacts. To ensure data quality, images with translational displacement beyond 3 mm or rotational deviation exceeding 3° were subsequently excluded. Subsequently, the corrected data volumes were spatially normalized to the standard Montreal Neurological Institute (MNI) coordinate space, preserving a voxel size of 3 × 3 × 3 mm^3^. Next, the spatially modulated images were smoothed with a 6 mm full width at half-maximal Gaussian kernel. Further preprocessing steps included detrending, regressing out covariates, and filtering (0.01–0.08 Hz).

### 2.4 Definition of regions of interest

**Figure 1:**
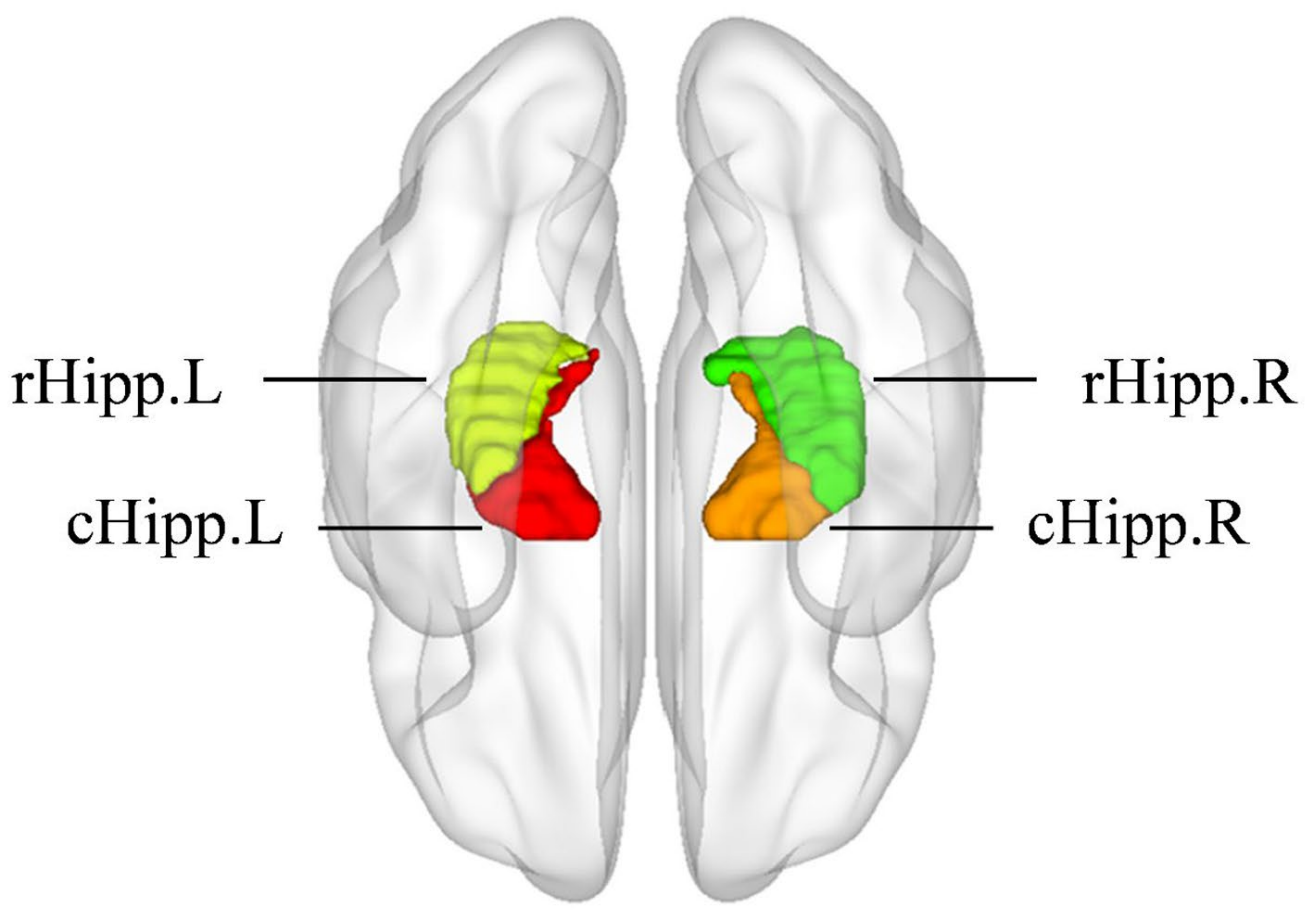
Illustration of four seed points in the left and right hippocampal subregions with distinct colors. rHipp, rostral hippocampus; cHipp, caudal hippocampus; L, left; R, right.

The bilateral rostral and caudal hippocampal regions were defined as regions of interest (ROIs), referencing the Brainnetome Atlas 246 (24, 25) (Figure 1).

### 2.5 Analysis of static and dynamic functional connectivity

The sFC analysis was quantified through Pearson’s correlation coefficients, measuring temporal correlations between voxel time courses within each ROI and the entire brain. Then, individual r-maps were normalized to Z-maps utilizing the Fisher Z-transformation to improve normality.

The dFC analysis was conducted using a sliding-window approach within the Temporal Dynamic Analysis toolkits of DPABI software, segmenting ROI time courses into short intervals of 30 TRs and a step width of 1 TR (16, 26). Research indicated that, under a Hamming-window approach for dFC estimation, a window duration in the range of approximately 30 s to 1 min is generally appropriate (21). Thus, we selected the sliding-window length to achieve a balance between capturing relatively rapid fluctuations in dynamic connectivity and obtaining more reliable correlation estimates by integrating information over a longer temporal segment. In total, 201 sliding windows of dFC were obtained. Within each sliding window, correlation maps were generated by calculating the temporal correlation coefficients between the ROIs and the corresponding time series of all other brain voxels. To enhance the normality of the correlation distribution, Fisher’s r-to-z transformation was applied to convert correlation maps into z-value maps. Subsequently, the dFC map was derived by computing the standard deviation across the 201 sliding windows. To evaluate potential biases induced by window width, we also repeated the analysis with a smaller window width of 20 TRs and a larger window width of 40 TRs (Supplementary Table S1; Supplementary Figure S1).

### 2.6 Statistical analysis

Demographic and clinical parameters were compared among the CM, EM and HC groups using the SPSS software (version 27.0, IBM Corp., Armonk, NY, USA). Sex distribution was analyzed using the chi-squared test, while age and education were evaluated by one-way analysis of variance. For clinical parameters, differences between EM and CM groups were evaluated with the Mann–Whitney U test. Regarding sFC and dFC analyses, voxel-wise group differences among the three groups were assessed through the analysis of the general linear model, controlling for age, sex, and education level. Multiple comparison correction was corrected with family-wise error theory (FWE, voxel-level *p* < 0.001, and cluster-level *p* < 0.05). Post-hoc tests were performed using R’s emmeans, with sidak multiple comparison correction. Cohen’s f^2^ was employed to quantify effect size for the GLM, and Cohen’s d was reported for the subsequent *post hoc* pairwise comparisons. Following Cohen’s interpretation, effect size could be characterized as approximately small, medium, and large when d ≈ 0.2/0.5/0.8 and at f^2^ ≈ 0.02/0.15/0.35, respectively (27). In subgroups of migraine, for the sFC and dFC values with significant between-group differences, spearman partial correlation analyses were conducted to examine their relationships with clinical parameters, while sex, age, and education level were served as covariates to mitigate potential confounding influences. The statistical significance threshold was set at *p* < 0.05.

## 3 Results

### 3.1 Demographic and clinical characteristics

Table 1 presents the demographic and clinical characteristics of the participants in this study. No significant differences were observed in gender (*p* = 0.139), age (*p* = 0.103), or education (*p* = 0.134) among the three groups. Patients with CM demonstrated higher headache frequency (*p* < 0.001), disease duration (*p* = 0.006), and VAS score (*p* = 0.034) compared to those with EM.

**Table 1:** Demographic and clinical characteristics.

Clinical features	EM (*n* = 52)	CM (*n* = 22)	HC (*n* = 67)	*p* value
Sex (male/female)	12/40	10/12	18/49	0.139^a^
Age (years)	40.12 ± 9.60	44.82 ± 15.74	44.96 ± 13.91	0.103^b^
Education level (years)	10.92 ± 3.95	9.32 ± 4.26	11.39 ± 4.32	0.134^b^
Headache frequency/month	3.84 ± 2.47	20.16 ± 4.52	-	<0.001^c^
Disease duration (years)	9.67 ± 6.91	16.09 ± 10.28	-	0.006^c^
VAS	6.81 ± 1.53	7.32 ± 1.58	-	0.034^c^

Continuous variables are presented as mean ± standard deviation.

EM, episodic migraine; CM, chronic migraine; HC, healthy control; VAS, Visual Analog Scales; *n*, number of subjects.

a *p* value obtained with Chi-square test.

b *p* value obtained with one-way analysis of variance.

c *p* value obtained with Mann–Whitney U test.

### 3.2 Inter-group differences in sFC and dFC

**Figure 2:**
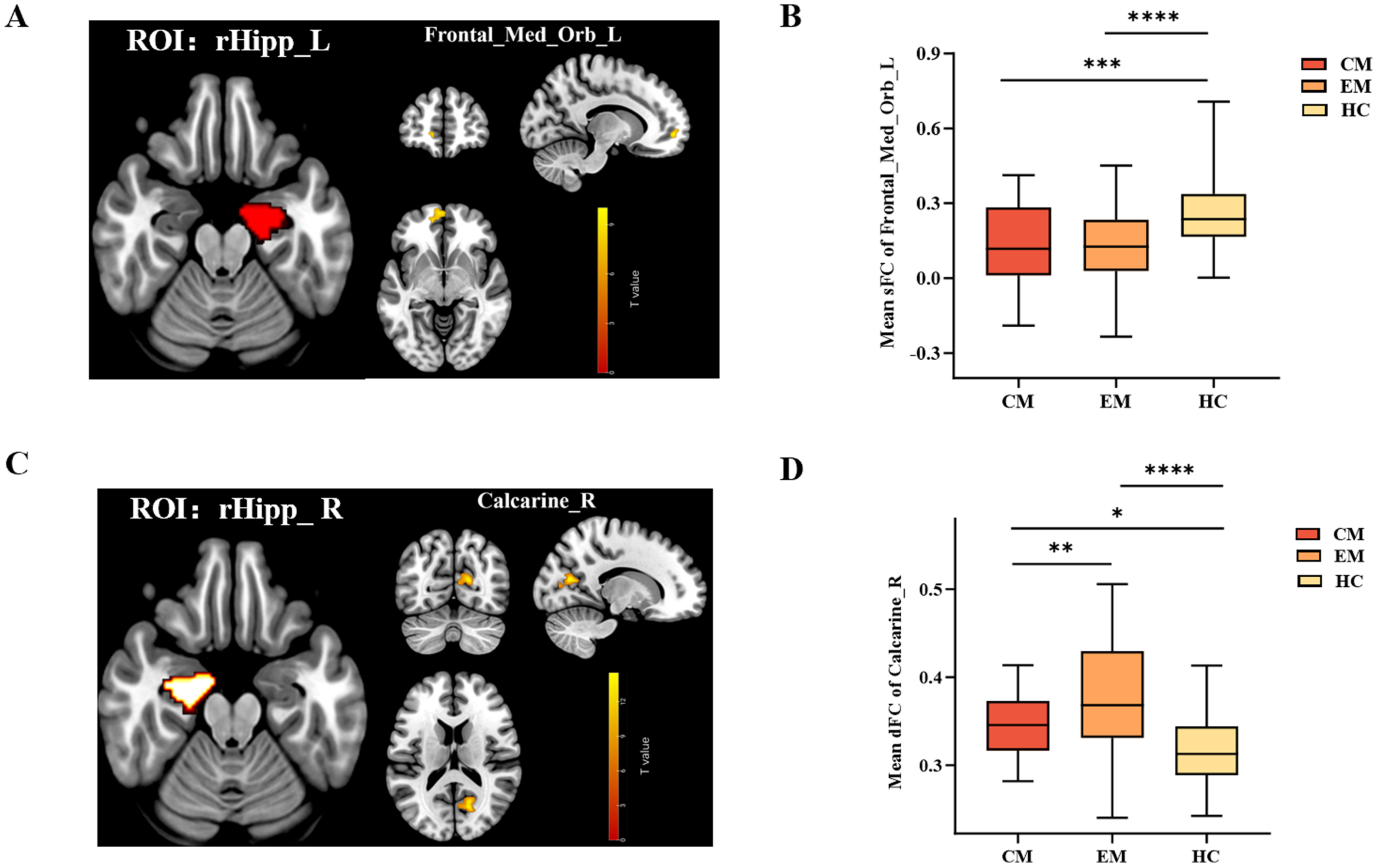
Intergroup differences in sFC and dFC strength among the CM, EM, and HC groups (seed: rHipp_L/rHipp_R). **(A,B)** With rHipp_L as the seed for sFC analysis, both EM and CM patients exhibited reduced sFC values between the rHipp_L and Frontal_Med_Orb_L compared with the HC, which may be associated with the pain perception and drug use. **(C,D)** With rHipp_R as the seed for dFC analysis, both migraine groups exhibited increased dFC values between the rHipp_R and calcarine_R in comparison with the HC group, with distinct differences observed between the EM and CM groups, which may be related to visual and emotional abnormalities. All results were thresholded at voxel-level *p* < 0.001 and cluster-level *p* < 0.05 (FWE corrected). Significant clusters identified by the general linear model were further examined using post-hoc pairwise tests implemented in R. sFC, Static functional connectivity; dFC, dynamic functional connectivity; rHipp, rostral hippocampus; Med, medial; Orb, orbital; L, left; R, right; EM, episodic migraine; CM, chronic migraine; HC, healthy control; FWE, family-wise error. *Statistically significant at the 0.05 level; **Statistically significant at the 0.01 level; ***Statistically significant at the 0.001 level; ****Statistically significant at the 0.0001 level.

As shown in Table 2 and Figure 2, the significant differences in sFC between the left rostral hippocampal (rHipp.L) and the left orbital part of medial frontal gyrus, as well as the differences in dFC between the right rostral hippocampal (rHipp.R) and the right calcarine among the three groups were identified in this research. Further post-hoc analysis indicated that compared to the HC group, both the CM (*p* = 0.0002, Cohen’s d = 0.935) and EM (*p* < 0.0001, Cohen’s d = 1.095) groups exhibited decreased sFC between the rHipp.L and the left orbital part of the medial frontal gyrus, while no significant differences in sFC within the hippocampal subregions were observed between the EM and CM groups. Regarding the dFC analysis, we found increased dFC between the rHipp.R and the right calcarine in patients with CM (*p* = 0.0280, Cohen’s d = 0.546) and EM (*p* < 0.0001, Cohen’s d = 1.238) compared to the HCs, and the CM group exhibited lower dFC between the rHipp.R and the right calcarine compared to the EM group (*p* = 0.0070, Cohen’s d = 0.696). When using window widths of 20 TRs and 40 TRs, the results remained highly consistent with the main findings (Supplementary Table S1; Supplementary Figure S1). In this study, abnormal FC between the cHipp and other brain regions was not observed.

**Table 2:** Differences in functional connectivity between bilateral hippocampal subregions and other brain regions among the three groups.

ROI	Brain region	Cluster size	Peak MNI coordinate			*F* value	Cohen’s f^2^
			x	y	z		
sFC							
rHipp_L	Frontal_Med_Orb_L	40	−12	54	−6	10.702	0.157
dFC							
rHipp_R	Calcarine_R	61	15	−69	15	14.741	0.217

All clusters were analyzed with a threshold of *p* < 0.001 and corrected for FWE at the cluster level to *p* < 0.05.

MNI, Montreal Neurological Institute; ROI, region of interest; sFC, static functional connectivity; dFC, dynamic functional connectivity; rHipp, rostral hippocampus; Med, Medial; Orb, orbital; L, left; R, right; FWE, family-wise error.

### 3.3 Correlation analysis

No correlations were observed between sFC and dFC values and clinical characteristics (i.e., frequency of headaches, duration of the disease, and VAS scores) in the migraine cohort.

## 4 Discussion

The present study utilized both sFC and dFC approaches to investigate hippocampal subfield connectivity alterations among the CM, EM and HC groups. In the sFC analyses, the EMs and CMs demonstrated reduced connectivity between the rHipp.L and the left orbital part of medial frontal gyrus as compared with HCs. In the dFC analysis, both the CM and EM groups exhibited increased connectivity between the rHipp.R and the right calcarine compared to HCs, and the CM group demonstrated decreased dFC between the aforementioned brain regions relative to the EM group. These findings partly supported our hypothesis regarding FC changes in particular hippocampal subregions of EM and CM patients and further revealed their inter-group differences. Insights gained from mapping intrinsic brain connectivity may provide a potentially mechanistic framework for understanding aspects of human behavior and mental disorders (28–32). The findings in our study may contribute to a deeper understanding of the potential pathological mechanisms underlying migraine chronification from the perspective of hippocampal subfield functional connectivity.

It is worth noting that the abnormal FC of hippocampal subregions among the EM, CM and HC groups in this study was mainly located in the rHipp rather than cHipp. One of the possible reasons is that the rHipp mainly participates in emotion regulation and affective processing, whereas the cHipp is more involved in spatial learning (15). Research on dFC further supported the important role of the rHipp in emotion regulation (33). Given the frequent emotional disturbances in migraine patients and their increased likelihood of being diagnosed with depression (34), which further indicated that the observed alterations in FC may be more concentrated in rHipp-related pathways involved in emotional processing. In addition, Holden et al. found a potential role for the rHipp in chronic pain (35), which was partially consistent with our findings in CM patients, suggesting the involvement of this brain region in the physiological mechanisms underlying migraine chronification through its connectivity with other brain areas. According to a meta-analysis of fMRI studies, the rHipp is primarily associated with the dorsal attention network, which is a complex cognitive network responsible for the processing of external information (36). Since co-occurring cognitive impairment problems are also common among migraineurs, the altered connectivity in the rHipp may be related to damage in the associated network, leading to cognitive deficits (37).

Our study revealed the reduced sFC between the rHipp and the left orbital part of the medial frontal gyrus in the CM and EM groups compared with the HC group. The orbitofrontal cortex (OFC) is a highly heterogeneous brain region involved in sensory integration, regulation of visceral responses, and emotional expression (38). As reported in the previous research, patients with CM exhibited irreversible hypometabolic changes in the OFC, which may contribute to the repeated abuse of medication and reveal its relationship with medication-overuse headache (39). Additionally, a voxel-based morphometry analysis indicated the reduction in gray matter in this region as a predictor of poor response to treatment (40). Consequently, the reduced connectivity of the OFC observed in this study may be asscociated with an increased vulnerability to drug abuse, potentially leading to the migraine chronification. This issue is particularly vital for patients with CM, who frequently experience medication-overuse headache with poorer quality of daily life (4). Future migraine research could focus on uncovering the core mechanisms by which the orbitofrontal cortex regulates pain processing and evaluating its potential as a biological marker for predicting treatment response, which would provide key scientific support for developing precise intervention strategies.

Moreover, the patients with migraine exhibited increased dFC between the rHipp and the calcarine cortex compared to the HCs. The medial temporal lobe including the hippocampus, is vital for goal-directed behavior, with a distinct population of target-selective neurons discovered in visual search tasks (41). Particularly, the rostral part of the hippocampal subfield has been demonstrated to exhibit a significant increase in neural activation in response to visual stimuli, indicating its core role in processing the novelty of visual stimuli (42). As most of the visual cortex is concealed within it and serves as a hub for visual pathways, the calcarine cortex participates in the perception of visual spots and the integration of various visual cues, thereby aiding in determining the target location of eye movements (43). In addition, the enhanced FC within the visual network suggests that increased visual stimuli may amplify nociceptive inputs, potentially leading to heightened pain perception (18). In the previous research, the increased changes in connectivity between the calcarine cortex and other brain regions along with its positive correlation to the severity of headache, have been observed in patients with migraine, suggesting the involvement of this brain region in pain perception (44, 45). Consistent with above studies, our study further revealed increased dFC between the calcarine cortex and rHipp in migraine patients. As mentioned in the previous diagnosis, photophobia is a common accompanying symptom among migraine patients (2). Consequently, the abnormal FC mentioned in this study may be related to visual dysfunction in those patients and exacerbate their pain levels.

Notably, in contrast to the patients with EM, CM patients with more frequent attacks showed reduced dFC between the calcarine cortex and the rHipp, which may relate to their visual abnormalities. Specifically, it manifests as impaired processing of visual motion speed in patients with higher frequency of migraine attacks (46). It is possible due to central sensitization caused by repeated chronic pain, leading to diminished perception of other sensory inputs and potentially resulting in visual processing anomalies with the visual motion speed (18, 47). Based on the above observations, the altered connectivity in CM patients is potentially linked to the mechanism of migraine chronification and may be related to pain hypersensitivity, as well as other sensory disturbances such as visual abnormalities. Therefore, personalized rehabilitation measures should be considered for patients to improve their sensory function and enhance their quality of life. It should be emphasized that our study did not directly assess medication use, emotional disorders, or visual symptom-related measures. Therefore, the interpretations linking orbitofrontal connectivity to medication use, hippocampal changes to emotional disorders and cognitive impairment, and calcarine connectivity to photophobia remain speculative. Future studies should directly quantify these clinical variables and further test whether the connectivity alterations observed here are associated with symptom severity or may predict treatment response.

Interestingly, no abnormal sFC values were found between the hippocampal subregions and other brain regions in CM and EM patients, whereas the significant difference in dFC was observed. Similar to parts of our study, previous research employing independent component analysis on changes in network connectivity between the CM and HC groups showed significant group differences in dynamic functional network connectivity analysis, but not in the static functional network connectivity analysis. It could be inferred that the functional characteristics of CM brains are dynamic rather than static, potentially experiencing fluctuations over time (18). The dFC method can explain how the brain quickly responds to and integrates external stimuli over different time scales and reveals changes in macro-neural activity patterns associated with cognition and behavior, but the sFC method is unable to capture these dynamic changes (21). Therefore, the group differences in dFC between CM and EM patients may help to understand the distinct changes exhibited by these two different types of migraines when faced with complex behavioral tasks, further revealing the abnormal activities in the associated brain regions. In summary, we believe that dFC may be more closely related to abnormal dynamic connectivity (48). The combination of sFC and dFC may provide a new perspective for understanding the mechanisms of migraine chronification. Moreover, no significant correlations between FC values and clinical parameters, aligning partially with previous studies (12, 49). This lack of association may reflect the complexity and heterogeneity of migraine symptoms: multiple interacting factors (such as pain-related characteristics and medication use) could obscure a direct linear relationship between FC changes and a single clinical measure across different disease states. In addition, connectivity changes may reflect mechanistic or state-related brain tissue properties that cannot be directly captured by cross-sectional clinical assessments. Finally, limited sample size and the cross-sectional study design may further reduce the ability to detect FC–clinical associations. Taken together, these considerations suggest that the clinical interpretability of our FC findings may require stratified analyses and longitudinal designs to better characterize the relationship between connectivity and migraine chronification.

Our research had several limitations. First, one limitation of this study is the relatively small and unequal sample size, particularly for CM group, which is due to its lower prevalence compared to EM in the general population. The overall smaller sample size increases the risk of Type II errors, meaning we may have missed some functionally connected hippocampal subregions with actually significant differences. This limitation should be considered when interpreting the findings, and future research with larger and more balanced samples is needed to confirm these results. Second, given that the participants were exclusively recruited from a single center, the results require further validation using a multicenter dataset to ensure their generalizability. Third, some patients had a history of using acute-phase therapeutic medications (e.g., non-steroidal anti-inflammatory drugs), but the specific types and dosages of these medications were not thoroughly recorded. This represents an important limitation that should be further explored and addressed in future research. Fourth, sliding-window dFC has certain limitations, mainly including dependence on window parameter selection and that apparent dynamics may partly reflect estimation noise. Finally, we must emphasize that our study is only a cross-sectional study, which restricts the inference of causal relationships based on the observed results. It is necessary to conduct longitudinal studies in the future to evaluate the causal link between FC outcomes and migraine chronification.

## 5 Conclusion

Altered sFC and dFC between the rHipp and the orbital part of medial frontal gyrus and calcarine in CM patients may be associated with dysfunctions in emotion, cognition, and multisensory integration, providing new insights into mechanisms of migraine chronification.

## Data Availability

The original contributions presented in the study are included in the article/Supplementary material, further inquiries can be directed to the corresponding authors.

## Footnotes

1
http://rfmri.org/dpabi

## Glossary

cHipp: Caudal hippocampus
CM: Chronic migraine
dFC: Dynamic functional connectivity
DPABI: Data Processing and Analysis for Brain Imaging
EM: Episodic migraine
FA: Flip angle
FOV: Field of view
FWE: Family-wise error theory
HC: Healthy control
L: Left
MNI: Montreal Neurological Institute
Med: Medial
OFC: Orbitofrontal cortex
Orb: Orbital
rHipp: Rostral hippocampus
rs-fMRI: Resting-state functional magnetic resonance imaging
ROI: Region of interest
R: Right
sFC: Static functional connectivity
TR: Repetition time
TE: Echo time
VAS: Visual Analog Scales
