# Supplementary material for: Disrupted functional connectivity in the hippocampal subregions of patients with migraine without aura: a functional study on mechanisms underlying migraine chronification
Source: Front Neurol. 2026 Jun 2;17:1821399. doi: 10.3389/fneur.2026.1821399 (PMC13268935; doi:10.3389/fneur.2026.1821399)
Supplement: Supplementary file 1 [file Table_1.docx]

**Table S1**. Comparison of dFC among the EM, CM and HC groups with window widths of 20 TRs and 40 TRs.

| **ROI** | **Brain region** | **Cluster size** | **Peak MNI coordinate** | | | **F value** | **Cohen’s f^2^** | **Post-hoc** |
| --- | --- | --- | --- | --- | --- | --- | --- | --- |
|  |  |  | **x** | **y** | **z** |  |  |  |
| **dFC (20 TRs)** |  |  |  |  |  |  |  |  |
| rHipp_R | Calcarine_R | 48 | 18 | -72 | 12 | 14.558 | 0.214 | EM>CM  (P=0.0082, Cohen’s d=0.683)  CM>HC  (P=0.0195, Cohen’s d=0.581)  EM>HC  (P<0.0001, Cohen’s d=1.256) |
| **dFC (40 TRs)** |  |  |  |  |  |  |  |  |
| rHipp_R | Calcarine_R | 24 | 15 | -63 | 15 | 11.948 | 0.176 | EM>CM  (P=0.0320, Cohen’s d=0.551)  CM>HC  (P=0.0448, Cohen’s d=0.450)  EM>HC  (P<0.0001, Cohen’s d=1.046) |

Note: All clusters were analyzed with a threshold of P<0.001 and corrected for FWE at the cluster level to P<0.05. Cohen’s f^2^/d represents the effect size.

dFC, dynamic functional connectivity; EM, episodic migraine; CM, chronic migraine; HC, healthy control; ROI, region of interest; MNI, Montreal Neurological Institute; TR, repetition time; rHipp, rostral hippocampus; R, right; FWE, family-wise error.


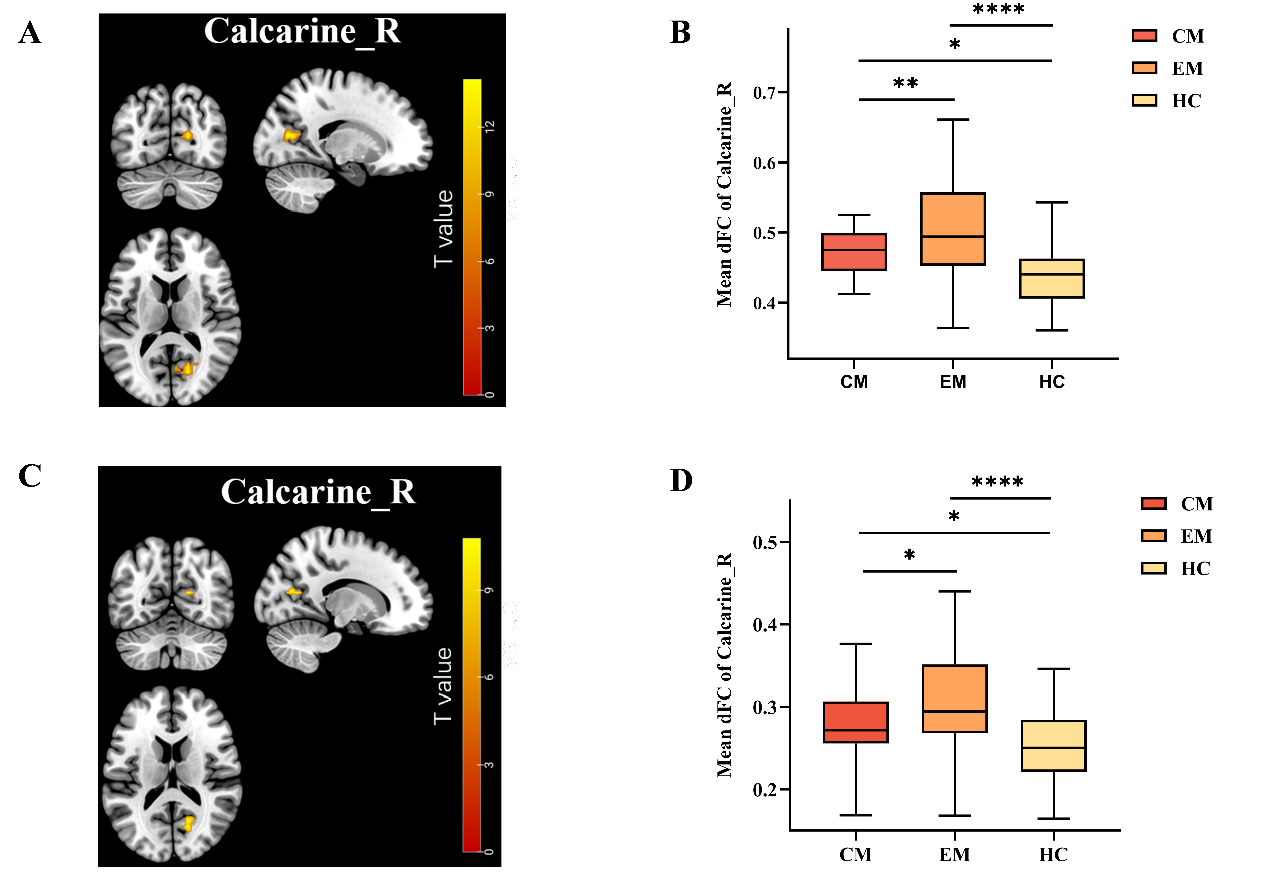


**Fig. S1.** Comparison of rostral hippocampal dFC values among the three groups with window widths of 20 TRs and 40 TRs. When the window widths were set to 20 TRs (A, B) or 40 TRs (C, D), altered dFC between the rHipp_R and the right calcarine was found among the three groups. Compared with HCs, both EM and CM exhibited increased dFC between these regions, and EM patients showed higher dFC than CM patients. All results were thresholded at voxel-level P<0.001 and cluster-level P<0.05 (FWE corrected). Significant clusters identified by the GLM were further examined using post-hoc pairwise tests implemented in R.

dFC, dynamic functional connectivity; rHipp, rostral hippocampus; R, right; EM, episodic migraine; CM, chronic migraine; HC, healthy control; TR, repetition time; GLM, general linear model; FWE, family-wise error.

*Statistically significant at the 0.05 level; **Statistically significant at the 0.01 level; ****Statistically significant at the 0.0001 level
